# Investigation of INDEL variants in apoptosis: the relevance to gastric cancer

**DOI:** 10.1186/s12881-020-01138-3

**Published:** 2020-10-19

**Authors:** Giovanna Chaves Cavalcante, Milene Raiol de Moraes, Cristina Maria Duarte Valente, Caio Santos Silva, Antônio André Conde Modesto, Paula Baraúna de Assumpção, Paulo Pimentel de Assumpção, Sidney Santos, Ândrea Ribeiro-dos-Santos

**Affiliations:** 1 Laboratório de Genética Humana e Médica, Programa de Pós-Graduação em Genética e Biologia Molecular, Universidade Federal do Pará, Av. Augusto Correa 01, Belém, Pará 66075-110 Brazil; 2 Núcleo de Pesquisas em Oncologia, Programa de Pós-Graduação em Oncologia e Ciências Médicas, Universidade Federal do Pará, Belém, Pará 66073-005 Brazil

**Keywords:** Gastric Cancer, Apoptosis, INDEL, FADD

## Abstract

**Background:**

Apoptosis is a type of cell death involved in different pathways inherent to the cell and the evasion from this mechanism has been related to cancer, although this process remains not very well comprehended. Gastric cancer (GC) is one of the most incident and aggressive types of cancer worldwide. In this study, we analyzed the distribution of INDEL variants in GC patients (Case) and individuals from the general population (Control) from the Amazon region, in which GC is remarkably frequent.

**Methods:**

A panel of nine INDEL markers in apoptosis-related genes (*BCL2* rs11269260, *CASP3* rs4647655, *CASP8* rs3834129 and rs59308963, *CASP9* rs4645982 and rs61079693, *FADD* rs4197, *FAS* rs10562972 and *TP53* rs17880560) was developed and genotyped by multiplex PCR in both groups.

**Results:**

In our analyses, only marker rs4197 (*FADD* gene) was associated to GC development as follows: INS/DEL genotype of rs4197 increasing in about 2-fold the chances of developing this type of cancer (*P* = 0.046; OR = 1.940; 95%CI = 1.011–3.725).

**Conclusion:**

Our results suggest that rs4197 (*FADD* gene) might play a role in gastric carcinogenesis in the investigated population. More studies are needed to clarify this relation. Here, we highlight the importance of investigating INDEL variants in genes involved in apoptosis.

## Background

Apoptosis is a type of programmed cell death (PCD) that may occur as a response to intra- or extracellular stimuli, leading to intrinsic pathway or extrinsic pathway, respectively. These are independent pathways that converge to the executor phase, the final stage of this death process [1–3]. Several genes are involved in apoptosis, out of which we highlight: *BCL2*, *CASP9* in the intrinsic pathway; *FAS*, *FADD*, *CASP8* in the extrinsic pathway; *TP53* in both pathways; and *CASP3* in the executor phase. All of these genes are important to the apoptotic mechanism, so that variants affecting their functions might dysregulate cell homeostasis and contribute to tumor development [4]. Although the apoptosis process is still not completely comprehended, this type of cell death mechanism has been related to cancer.

Gastric cancer (GC) is one of the most frequent and aggressive types of cancer worldwide, usually presenting an unfavorable prognosis [5, 6]. In Brazil, GC is the sixth most incident and the fifth most mortal type of cancer [6]. Brazil is a country with a highly admixed population, composed mainly of European, African, Amerindian and Asian ancestry. Many studies in this country have highlighted differences between these ancestries and the Brazilian regions [7–11], including aspects of cancer development [12]. This is also notable by the higher incidence of GC in northern Brazil in comparison to the general rates of this country and the world [13].

Therefore, in this study, we aimed to analyze the distribution of nine Insertion/Deletion (INDEL) polymorphisms in apoptosis-related genes in gastric cancer patients and cancer-free individuals from the general population of the Amazon region.

## Methods

### Sampling

Case group was composed of 93 individuals with gastric cancer diagnosis from Pará state and control group was composed of 98 cancer-free individuals of the general population from the same state, located in the Amazon region of Brazil. From each individual, blood or buccal swab samples were collected. All participants signed an informed consent, with approval by the Committee for Research Ethics of Hospital João de Barros Barreto under Protocol Number: 64399617.7.0000.5634.

### DNA extraction and quantification

DNA extraction was performed with phenol-chloroform method, based on [14]. Quantification of DNA was performed with NanoDrop 1000 and Qubit™ (Thermo Fisher Scientific, Wilmington, DE, USA).

### Selection of INDEL markers

The markers were selected according to the main criteria: (i) they must be in genes involved in apoptosis pathways; (ii) they must be INDEL-type polymorphisms, hence presenting the potential to modify the function of the protein; (iii) they must have minor allele frequency (MAF) ≥ 10%. Table 1 shows the technical features of the investigated markers.

**Table 1:** Technical characterization of the markers included in the panel

Gene	ID	Region	Alleles	MAF	***Primers***	***Amplicon***
*BCL2*	rs11269260	Intron	TCTATCACCGATCATT/−	0.37	F5’GCTTCCAGTTCCATCCATGT3’ R5’CTCAGCGTGGTAGTGTTGGA3’	189–205
*CASP3*	rs4647655	Intron	−/AAATCCTGAA	0.28	F5’AGGAGTATCCCCTCGTGGAC3’ R5’CAAGAGTCAGGCAAAAACAGG3’	379–389
*CASP8*	rs3834129	Promoter	AGTAAG/−	0.39	F5’CTCTTCAATGCTTCCTTGAGGT3’ R5’CTGCATGCCAGGAGCTAAGTAT3’	249–255
*CASP8*	rs59308963	Intron	ATTCTGTC/−	0.26	F5’TTTTTGTCCTCCAAGCTTCC3’ R5’GAACAAGAGAGAGGGCAGGA3’	261–269
*CASP9*	rs4645982	Intron	−/TCCCCGCACTGACCTCACG	0.42	F5’GGTGACCCCAGAATTGACCCT3’ R5’GCCCTCAGGACGCACCTCTG3’	336–353
*CASP9*	rs61079693	Intron	AAAA/−	0.32	F5’CATGCACAGCTATCCAGGAG3’ R5’TTGTTCCTGTCCGATAGATGC3’	458–462
*FADD*	rs4197	3′ UTR	−/TGT	0.47	F5’TGCCCCTACTTAGCAGTCTCA3’ R5’GAGAGGTGGAGAACTGGGATT3’	278–281
*FAS*	rs10562972	Intron	TTC/−	0.12	F5’GCATCAGGACGCTGAACATA3’ R5’AATGCAACTTGCTCCAGAGG3’	368–371
*TP53*	rs17880560	3′-Flanking	−/GCCGTG	0.21	F5’CTGTGTGTCTGAGGGGTGAA3’ R5’ATCCTGCCACTTTCTGATGG3’	400–406

### Genotyping

The panel of markers described above was genotyped by Multiplex PCR, allowing the amplification of all of these markers in a single reaction, followed by capillary electrophoresis and fragment analysis. Protocol preparation of PCR samples was: 5.0 μL of QIAGEN Multiplex PCR Master Mix, 1.0 μL of Q-solution, 2.0 μL of water, 1.0 μL of primer mix and 1.0 μL of DNA per sample. Amplification reaction was done using Veriti thermal cycler (Thermo Fisher Scientific) with the following protocol: 95 °C for 15 min, followed by 35 cycles of 94 °C for 45 s, 60 °C for 90 s and 72 °C for 1 min, with a final extension at 70 °C for 30 min. The protocol for capillary electrophoresis was: 1.0 μL of PCR product, 8.5 μL of HI-DI deionized formamide and 0.5 μL of GeneScan 500 LIZ pattern size standard (reagents by Thermo Fisher Scientific). DNA fragments were separated and analyzed using ABI PRISM 3130 genetic analyzer and GeneMapper ID v.3.2 software (both by Thermo Fisher Scientific).

In addition to the panel of apoptosis markers, a panel of 61 ancestry informative markers (AIM) previously developed and expanded by our research group was also employed in this study, following the established protocols [15, 16]. This was done considering that the Brazilian population is highly admixed in terms of genetic contributions from different parental populations, mainly European, African and Native American. In order to control the possible influence of these ancestries in the genotypic distribution, leading to misinterpretation, we have used this AIM panel, composed of ancestry-specific markers that are able to estimate individual and population genetic contribution.

### Statistical analysis

Inference of genetic ancestry based on the AIM panel was done with Structure software v.2.3.4 [17]. R language [18] was used to assess Hardy-Weinberg Equilibrium (HWE) of genotype distribution. JASP software v. 0.9.2.0 [19] was used to perform all other statistical analyses (Student’s *t* test, chi-squared test, logistic regression). *P*-values ≤0.05 were considered statistically significant.

## Results

Firstly, we assessed HWE for genotypic distribution and found that only two markers, rs4645982 (*CASP9*) and rs4197 (*FADD*), deviated from HWE in case group (*P* = 0.0035 and *P* = 0.0026, respectively).

Then, we compared sex, age and controlled genetic ancestry of case and control groups in order to verify possible confounding factors. In addition, staging (I-IV) of patients was assessed. These data are described in Table 2.

**Table 2:** Demographic data for case (gastric cancer patients) and control groups

Variable	Case	Control	***P***-value
N	91	95	
Age, years^a^	42.15 ± 1.66	32.18 ± 1.17	< 0.001
Sex, % of male/female^b^	48.9/51.1	50.0/50.0	1.000
European ancestry^c^	0.541 ± 0.028	0.587 ± 0.021	0.251
African ancestry^c^	0.152 ± 0.018	0.124 ± 0.013	0.966
Native American ancestry^c^	0.307 ± 0.022	0.289 ± 0.019	0.649
Stage I, %	3.3	NA	0.241
Stage II, %	20.7	NA	
Stage III, %	26.0	NA	
Stage IV, %	50.0	NA	

^a^Values are expressed as mean ± SE (Standard Error of Mean), Student’s t-test; b ^c^Values are expressed as distribution percentages, chi-squared test; ^c^Values are expressed as mean ± SE, Mann-Whitney test. *NA* Not Applicable

In these analyses, only age presented a statistically significant difference and it was included for correction in the panel analysis. It is noteworthy that 53% of the patients included in case group presented diffuse-type adenocarcinoma, while 47% presented intestinal-type adenocarcinoma, and the observed mean age (mean age ± SE) for these subtypes was 39.20 ± 1.397 and 44.34 ± 1.526, respectively, so that the mean age for case group as a whole was lower than it is usually observed for patients with GC in the world.

Nevertheless, in the analysis performed for each genotype (carriers of a genotype vs. non-carriers of such genotype as a reference group), only INS/DEL genotype of rs4197 (*P* = 0.046; OR = 1.940; 95%CI = 1.011–3.725) presented significant association to risk of developing GC in the investigated population (Table 3).

**Table 3:** Genotypic distribution of the investigated variants for patients with GC in comparison to control group. *P*-value, OR and 95%CI were obtained with logistic regression adjusted for age, which was done for the genotype as reference in each line (DEL/DEL vs. the other genotypes of that marker, and the same for INS/DEL and INS/INS)

Gene	Genotype	Case (%)*	Control (%)*	***P***-value	OR (95%CI)
*BCL2*	rs11269260	93	98		
	DEL/DEL	13 (14)	19 (19.4)	0.844	1.090 (0.463–2.567)
	INS/DEL	51 (54.8)	42 (42.9)	0.149	1.589 (0.848–2.977)
	INS/INS	29 (31.2)	37 (37.8)	0.092	0.556 (0.281–1.101)
*CASP3*	rs4647655	93	96		
	DEL/DEL	47 (50.5)	48 (50.0)	0.898	0.959 (0.511–1.802)
	INS/DEL	39 (41.9)	39 (40.6)	0.995	1.002 (0.528–1.904)
	INS/INS	7 (7.5)	9 (9.4)	0.825	1.136 (0.365–3.537)
*CASP8*	rs3834129	93	98		
	DEL/DEL	27 (29.0)	19 (19.4)	0.098	1.851 (0.893–3.835)
	INS/DEL	38 (40.9)	42 (42.9)	0.987	0.995 (0.526–1.880)
	INS/INS	28 (30.1)	37 (37.7)	0.132	0.596 (0.303–1.169)
	rs59308963	89	93		
	DEL/DEL	30 (33.7)	29 (31.2)	0.749	1.116 (0.568–2.195)
	INS/DEL	41 (46.1)	44 (47.3)	0.727	0.893 (0.471–1.690)
	INS/INS	18 (20.2)	20 (21.5)	0.953	1.024 (0.467–2.244)
*CASP9*	rs4645982	81	58		
	DEL/DEL	16 (19.8)	13 (22.4)	0.897	0.943 (0.392–2.272)
	INS/DEL	24 (29.6)	20 (34.5)	0.583	0.808 (0.377–1.732)
	INS/INS	41 (50.6)	25 (43.1)	0.535	1.254 (0.613–2.567)
	rs61079693	89	94		
	DEL/DEL	22 (24.7)	26 (27.7)	0.418	0.742 (0.360–1.529)
	INS/DEL	46 (51.7)	44 (46.8)	0.765	1.102 (0.585–2.076)
	INS/INS	21 (23.6)	24 (25.5)	0.621	1.209 (0.570–2.563)
*FADD*	rs4197	93	97		
	DEL/DEL	31 (33.3)	41 (42.3)	0.099	0.573 (0.295–1.111)
	INS/DEL	57 (61.3)	48 (49.5)	0.046	1.940 (1.011–3.725)
	INS/INS	5 (5.4)	8 (8.2)	0.441	0.600 (0.163–2.202)
*FAS*	rs10562972	89	93		
	DEL/DEL	1 (1.1)	0 (0.0)	0.988	991,452.554 (0.000–∞)
	INS/DEL	10 (11.2)	21 (22.6)	0.260	0.603 (0.250–1.454)
	INS/INS	78 (87.6)	72 (77.4)	0.329	1.535 (0.650–1.125)
*TP53*	rs17880560	87	93		
	DEL/DEL	57 (65.5)	58 (62.4)	0.738	1.119 (0.579–2.166)
	INS/DEL	26 (29.9)	31 (33.3)	0.714	0.880 (0.445–1.741)
	INS/INS	4 (4.6)	4 (4.3)	0.964	1.035 (0.232–4.623)

*Sample number; *P*-value: *P*-value adjusted for age, *OR* Odds Ratio, *CI* Confidence Interval

## Discussion

In the last decade, it has been established that tumors display different abilities in order to grow, survive and proliferate, of which we highlight here resistance to cell death, including apoptosis [20]. However, there is still a lot to discover about these mechanisms.

Apoptosis is a complex process that involves the protein action of several different genes, such as *BCL2*, *CASP3*, *CASP8*, *CASP9*, *FADD*, *FAS* and *TP53*. Dysfunction in these genes may lead to deregulation of cell death and, thus, tumor development. Therefore, in this study, we investigated whether nine INDEL variants in the mentioned genes might influence gastric carcinogenesis, by comparing their distribution in GC patients and cancer-free individuals.

Regarding the observed deviation from HWE in the distribution of rs4197 and rs4645982 in case group, no previous studies were found with these markers, but their genotype distribution varies greatly between different populations in 1000 Genomes Project database [21]. Curiously, their distribution also presented a deviation from HWE in most populations in that database, suggesting that there could be a selective advantage leading to this pattern and/or that the deviation observed here could be due to population substructure, especially considering the relatively recent admixture process in Brazil, as previously observed for other markers in this population [7, 22]. As such, it is an expected process in admixed populations, and it could even highlight the potential of these markers. In fact, it has been suggested that HWE deviation only in patient’s group could support possible locus-disease associations [23].

Then, we carried on to the analyses performed for each genotype (carriers of a genotype vs. non-carriers of such genotype as a reference group) in cases and controls. In these analyses, our results suggest that individuals carrying INS/DEL of rs4197 have about 2-fold chances of developing GC than non-carriers (*P* = 0.046; OR = 1.940; 95%CI = 1.011–3.725).

To the best of our knowledge, this is the first study investigating variant rs4197. It is a 3-bp INDEL in the 3′ UTR of *FADD* gene, a key adaptor molecule that transmits death signals from death receptors during extrinsic apoptosis, thus being crucial to a variety of processes [24]. In fact, it seems that it could affect protein features due to the importance of 3′-UTR [25]. Depletion of FADD protein action may lead to failure of apoptosis and, thus, to tumor development. Absence of FADD expression has been suggested as marker of tumor development in mice and cancer prognosis in humans, due to the involvement of this protein with cell apoptosis, survival and proliferation [26]. Therefore, investigation of *FADD* gene should help the understanding of cancer development, including gastric carcinogenesis.

As for the variants that did not present a statistical association here, this may suggest that they either do not play a role in gastric carcinogenesis or it is a population-specific treat. Indeed, some of these variants have been associated cancer development in a few studies, but not in others. For instance, one of these variants (rs3834129 in *CASP8*) has been previously studied by our research group in other sampling of the same region: we have associated INS/INS genotype of this variant to reduced chances of developing B-cell acute lymphoblastic leukemia [27], but have not found any association of this variant with GC or colorectal cancer [22], so that our study corroborates the findings in the latter. Thus, the association of such variants to GC and other types of cancer is still a matter of great discussion.

## Conclusions

Despite a few limitations (sample number and average age of both groups), this study contributed to an increased knowledge on variants in apoptosis-related genes in regard to GC development. As future perspectives, we recommend studies in the same population with a greater sample number and in different populations for comparison, as well as functional studies focused on the proteins to possibly reinforce the involvement of *FADD* gene in gastric carcinogenesis. Although such future studies are recommended to strengthen our results, this work contributes to a better understanding of these genes and INDEL variants in regard to gastric cancer.

## Data Availability

The datasets generated and/or analyzed during the current study are available in the Figshare repository (10.6084/m9.figshare.13040078).

## Abbreviations

AIM: Ancestry Informative Marker
BCL2: BCL2 apoptosis regulator
CASP3: Caspase 3
CASP8: Caspase 8
CASP9: Caspase 9
CI: Confidence Interval
DEL: Deletion
DNA: Deoxyribonucleic Acid
FADD: Fas associated via death domain
FAS: Fas cell surface death receptor
GC: Gastric Cancer
HWE: Hardy-Weinberg Equilibrium
ID: Identification
INDEL: Insertion/Deletion
INS: Insertion
MAF: Minor Allele Frequency
OR: Odds Ratio
PCD: Programmed Cell Death
PCR: Polymerase chain reaction
TP53: Tumor protein p53
UTR: Untranslated Region

## References

[1] Elmore S (2007). Apoptosis: a review of programmed cell death. Toxicol Pathol.

[2] Galluzzi L, Vitale I, Aaronson SA, Abrams JM, Adam D, Agostinis P (2018). Molecular mechanisms of cell death: recommendations of the nomenclature committee on cell death 2018. Cell Death Differ.

[3] Kerr JFR, Wyllie AH, Currie AR (1972). Apoptosis: a basic biological phenomenon with Wideranging implications in tissue kinetics. Br J Cancer.

[4] Cavalcante GC, Schaan AP, Cabral GF, Santana-da-Silva MN, Pinto P, Vidal AF (2019). A Cell’s fate: an overview of the molecular biology and genetics of apoptosis. Int J Mol Sci.

[5] Pinheiro D (2014). Do R, Ferreira WAS, Barros MBL, Araújo MD, Rodrigues-Antunes S, Borges B do N. perspectives on new biomarkers in gastric cancer: diagnostic and prognostic applications. World J Gastroenterol.

[6] GLOBOCAN (2018). Cancer today.

[7] Amador MAT, Cavalcante GC, Santos NPC, Gusmão L, Guerreiro JF, Ribeiro-dos-Santos Â, et al. Distribution of allelic and genotypic frequencies of IL1A, IL4, NFKB1 and PAR1 variants in Native American, African, European and Brazilian populations. BMC Res Notes. 2016;9(1) [cited 2019 Feb 21] Available from: http://www.biomedcentral.com/1756-0500/9/101. 10.1186/s13104-016-1906-9 PMC4754858 26879815

[8] Benedet AL, Moraes CF, Camargos EF, Oliveira LF, Souza VC, Lins TC (2012). Amerindian genetic ancestry protects against Alzheimer’s disease. Dement Geriatr Cogn Disord.

[9] Brum DG, Luizon MR, Santos AC, Lana-Peixoto MA, Rocha CF, Brito ML (2013). European ancestry predominates in neuromyelitis optica and multiple sclerosis patients from Brazil. PLoS One.

[10] Rolim H, Cronemberger S, Rangel H, Batista WD, Bastos-Rodrigues L, De Marco L (2016). The role of genetic ancestry in Brazilian patients with primary congenital Glaucoma. J Glaucoma.

[11] Souza MCLA, Martins CS, Silva-Junior IM, Chriguer RS, Bueno AC, Antonini SR (2014). NR3C1 polymorphisms in Brazilians of Caucasian, African, and Asian ancestry: glucocorticoid sensitivity and genotype association. Arq Bras Endocrinol Metabol.

[12] da Silva EM, Fernandes MR, de Carvalho DC, Leitao LPC, Cavalcante GC, Pereira EEB, et al. Effect of genetic ancestry to the risk of susceptibility to gastric cancer in a mixed population of the Brazilian Amazon. BMC Research Notes. 2017;10(1) [cited 2019 Feb 21] Available from: https://bmcresnotes.biomedcentral.com/articles/10.1186/s13104-017-2963-4. 10.1186/s13104-017-2963-4 PMC5707813 29187240

[13] INCA (2018). INCA - Instituto Nacional de Câncer – Estimativa. 2018. INCA - Instituto Nacional de Câncer.

[14] Sambrook J, Fritsch EF, Maniatis T (1989). Molecular cloning: a laboratory manual. Molecular cloning: a laboratory manual.

[15] Santos NPC, Ribeiro-Rodrigues EM, Ribeiro-dos-Santos ÂKC, Pereira R, Gusmão L, Amorim A (2010). Assessing individual interethnic admixture and population substructure using a 48-insertion-deletion (INSEL) ancestry-informative marker (AIM) panel. Hum Mutat.

[16] Andrade RB, Amador MAT, Cavalcante GC, Leitão LPC, Fernandes MR, Modesto AAC (2018). Estimating Asian Contribution to the Brazilian Population: A New Application of a Validated Set of 61 Ancestry Informative Markers. G3&amp;#58; Genes|Genomes|Genetics.

[17] Pritchard JK, Stephens M, Donnelly P (2000). Inference of population structure using multilocus genotype data. Genetics..

[18] Core R (2014). Team. R: a language and environment for statistical computing. R Foundation for statistical Computing.

[19] Team JASP (2018). JASP (Version 0.9).

[20] Hanahan D, Weinberg RA (2011). Hallmarks of cancer: the next generation. Cell..

[21] Auton A, Brooks LD, Durbin RM, Garrison EP, Kang HM, 1000 Genomes Project Consortium (2015). A global reference for human genetic variation. Nature..

[22] Cavalcante GC, Amador MA, Ribeiro Dos Santos AM, Carvalho DC, Andrade RB, Pereira EE (2017). Analysis of 12 variants in the development of gastric and colorectal cancers. World J Gastroenterol.

[23] Yu K-D, Di G-H, Fan L, Shao Z-M (2009). Test of hardy–Weinberg equilibrium in breast cancer case-control studies: an issue may influence the conclusions. Breast Cancer Res Treat.

[24] Tourneur L, Chiocchia G (2010). FADD: a regulator of life and death. Trends Immunol.

[25] Mayr C (2018). What are 3′ UTRs doing? Cold spring Harb Perspect biol.

[26] Tourneur L, Buzyn A, Chiocchia G (2005). FADD adaptor in cancer. Med Immunol.

[27] Carvalho DC, Wanderley AV, Amador MAT, Fernandes MR, Cavalcante GC, Pantoja KBCC (2015). Amerindian genetic ancestry and INDEL polymorphisms associated with susceptibility of childhood B-cell leukemia in an admixed population from the Brazilian Amazon. Leuk Res.

